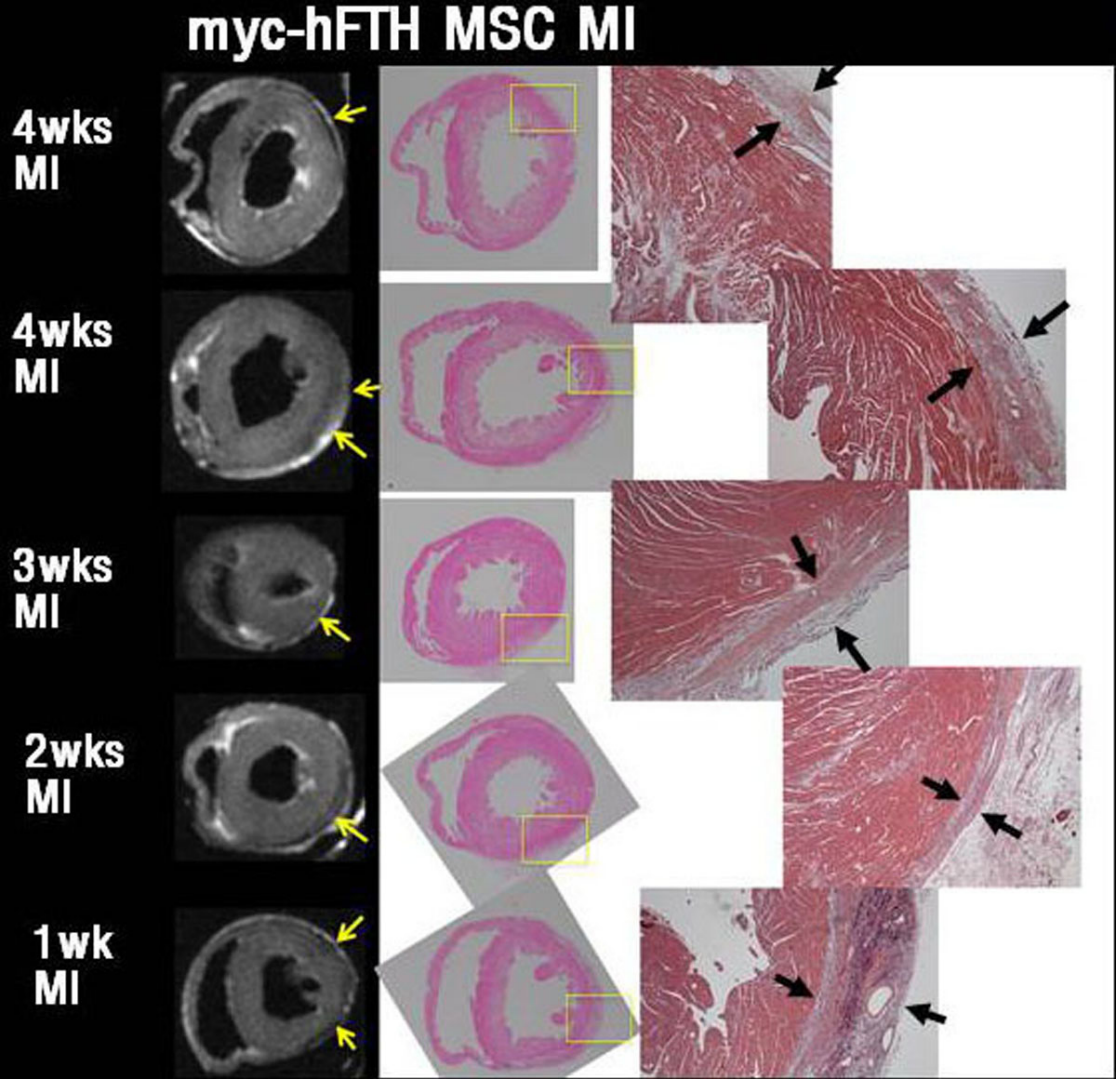# Ferritin as a reporter gene of *in vivo* stem cell tracking by 9.4-T cardiac MR in a rat model of myocardial infarction

**DOI:** 10.1186/1532-429X-15-S1-P31

**Published:** 2013-01-30

**Authors:** Eun-Ah Park, Whal Lee, Panki Kim, Hoe Suk Kim

**Affiliations:** 1 Radiology, Seoul National University Hospital, Seoul, Republic of Korea

## Background

The current methods utilized to track stem cells by cardiac MR are affected by important limitations, and new solutions are needed.

## Methods

We tested human ferritin heavy chain (hFTH) tagged with both myc and green fluorescence protein (GFP) as a reporter gene for in vivo tracking of stem cells by cardiac MR. Rat mesenchymal stem cells (rMSCs) were transduced with lentiviurs to overexpress hFTH. Myocardia infarction was induced by cryoinjury in rats, and the animals were immediately subjected to intramyocardial injection of 100 µl of 106 rMSCs (experiment group) or buffer solution (control group) in the border zone. *In vivo* cine and in vivo and ex vivo multiecho T2 weighted images were obtained by 9.4T cardiac MR.

## Results

Four-week follow-up cine MR showed that marked left ventricular remodeling developed in the control group. T2 relaxation time of in vivo and *ex vivo* images was significantly decreased in the infarct area compared to remote normal myocardium in the experiment group, but not in the control group. GFP and myc immune-staining confirmed the presence of differentiated rMSCs around infarct area in the experiment group.

## Conclusions

hFTH can be used as a MR reporter gene to track dividing and differentiating stem cells in the beating heart while simultaneously monitoring cardiac morpho-functional changes.

## Funding

This study was supported by a grant of the Korean Health Technology R&D Project, Ministry of Health & Welfare, Republic of Korea (No.A100131) and partly by the National Research Foundation of Korea (NRF) grant funded by the Korean government (MEST) (2011-0000174, 2011-0005381).

**Figure 1:**
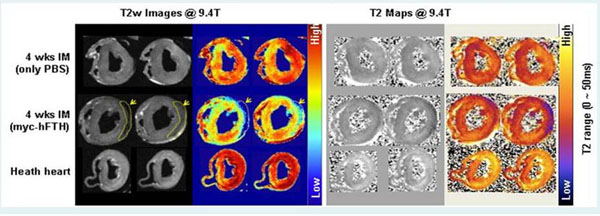


**Figure 2:**